# Directed Evolution of FLS2 towards Novel Flagellin Peptide Recognition

**DOI:** 10.1371/journal.pone.0157155

**Published:** 2016-06-06

**Authors:** Laura Helft, Mikayla Thompson, Andrew F. Bent

**Affiliations:** 1 Department of Plant Pathology, University of Wisconsin–Madison, Madison, Wisconsin, United States of America; 2 Cellular and Molecular Biology Program, University of Wisconsin–Madison, Madison, Wisconsin, United States of America; Virginia Tech, UNITED STATES

## Abstract

Microbe-associated molecular patterns (MAMPs) are molecules, or domains within molecules, that are conserved across microbial taxa and can be recognized by a plant or animal immune system. Although MAMP receptors have evolved to recognize conserved epitopes, the MAMPs in some microbial species or strains have diverged sufficiently to render them unrecognizable by some host immune systems. In this study, we carried out *in vitro* evolution of the *Arabidopsis thaliana* flagellin receptor FLAGELLIN-SENSING 2 (FLS2) to isolate derivatives that recognize one or more flagellin peptides from bacteria for which the wild-type *Arabidopsis* FLS2 confers little or no response. A targeted approach generated amino acid variation at FLS2 residues in a region previously implicated in flagellin recognition. The primary screen tested for elevated response to the canonical flagellin peptide from *Pseudomonas aeruginosa*, flg22. From this pool, we then identified five alleles of *FLS2* that confer modest (quantitatively partial) recognition of an *Erwinia amylovora* flagellin peptide. Use of this *Erwinia*-based flagellin peptide to stimulate *Arabidopsis* plants expressing the resulting *FLS2* alleles did not lead to a detectable reduction of virulent *P*. *syringae* pv. *tomato* growth. However, combination of two identified mutations into a single allele further increased FLS2-mediated responses to the *E*. *amylovora* flagellin peptide. These studies demonstrate the potential to raise the sensitivity of MAMP receptors toward particular targets.

## Introduction

Microbe-associated molecular patterns (MAMPs) are conserved and often relatively abundant molecular signatures that are present across a broad range of microbes (i.e., multiple genera within a kingdom or phylum), and trigger defense responses of plant and/or animal innate immune systems [1–3]. Examples of viral, oomycete, fungal, or bacterial MAMPs recognized by plants include double-stranded RNA, chitin, β-glucans, elongation factor Tu, and flagellin [2, 4, 5]. Different MAMPs trigger an overlapping suite of plant responses, although the amplitude and duration of these responses differ depending on the host and the particular MAMP stimulus [3, 6]. In approximate order from early to late responses, these responses can include ion fluxes, a reactive oxygen species (ROS) burst, MAP kinase activation, ethylene-mediated signaling, poly-(ADP)ribosylation changes, stomatal closure, transcriptional reprogramming, and callose deposition [2, 7–11]. Plant responses to MAMPs can reduce pathogen growth on the host [3, 12]. During extended exposure to MAMP molecules an inhibition of plant growth often occurs, and this growth/defense tradeoff is often used in the seedling growth inhibition assay, one of the most sensitive and convenient assays for FLS2 receptor activation [2, 13–15].

MAMP receptors often have extracellular repetitive protein domains that facilitate ligand recognition, that are coupled in plant MAMP receptors to an intracellular kinase domain for downstream signaling. Cloned and characterized MAMP receptors (also known as pattern recognition receptors or PRRs) from plants include the flagellin receptor FLS2 [16], the chitin co-receptors CEBiP, SERK1, LysM RLK1/CERK1 and LYK5 [17–21], the EF-Tu receptor EFR [22], and the xylanase receptors EIX1 and EIX2 [23], and others [3]. Xa21, Cf-9 and Ve-1are examples of R gene products that can now be jointly classified as both R gene products and MAMP receptors [3, 24]. MAMP receptors such as CEBiP and CERK1 contain extracellular LysM repeats, while FLS2, Xa21, SERK1, EIX1, EIX2, EFR and others have extracellular leucine-rich repeat (LRR) domains [2, 3].

LRRs are a spiral-shaped ligand interaction domain that can facilitate recognition of many types of molecules, including peptides, lipids, nucleic acids, and small molecule hormones [25–27]. Vertebrate TLRs also contain extracellular LRR domains for MAMP detection, and LRR domains are also present in most plant R gene products and a wide variety of other receptor proteins [25–27]. The β-strand, β-turn (concave) face of the LRR domain often mediates ligand interactions, although this is not universally true [27–29].

*Arabidopsis* FLAGELLIN-SENSING 2 (FLS2; [16]) is a MAMP receptor that recognizes flagellin or peptides based on the recognized flagellin epitope, such as the 22 amino acid flg22 peptide that matches a segment of *Pseudomonas aeruginosa* flagellin [30]. Orthologs of FLS2 have been cloned from diverse species including tomato, *Nicotiana benthamiana* and rice; this presence throughout the higher plant lineage may involve functional distinctions or constraints relative to MAMP receptors such as EFR and Xa21 that have been detected in only one plant family [31–35]. Numerous features of the FLS2 receptor and its interactions with other proteins have been defined [6]. This includes the recent solution of a co-crystal structure for the extracellular domains of FLS2 and the co-receptor BAK1, and the FLS2 peptide ligand flg22 [36]. Flagellin activates FLS2 via a two-part ‘address/message’ process in which the binding activity of flagellin peptides with respect to FLS2 is spatially and functionally separable from the signaling activation domains of those peptides [37, 38]. Flagellin is also a recognized MAMP of the mammalian immune system, but the TLR5 receptor of mammals recognizes a different flagellin region [39].

FLS2 can contribute to disease resistance. *Arabidopsis fls2*^*-*^ plants exhibit elevated susceptibility to some bacterial infections, and pre-treatment with flg22 can increase disease resistance in FLS2-expressing plants including *Arabidopsis* and rice [12, 40]. Similarly, constitutive expression of flagellin protein in rice plants increased resistance to *Magnaporthe grisea* [41]. FLS2 has been implicated as a key component of the non-host resistance of *Arabidopsis* to *Pseudomonas syringae* pv *phaseolicola* [42]. However, FLS2 does not play a detectable role in resistance to all bacterial plant pathogens. This may be due to bacterial evasion of detection through altered flagellin structure [30, 40, 43–47]. Glycosylation of flagellin may also be an important determinant of recognition by the host [48, 49]. Other factors include reduced flagellin expression, masking or degradation of flagellins, infection in tissues where FLS2 is less effective, or to effector-mediated suppression of FLS2 signaling [30, 43, 44, 50–55]; see also [56, 57]. Yet the successful transfer of EFR across genera [58] provides evidence that transgenic plants with novel MAMP receptors could be a broadly feasible method to expand disease control.

Although MAMPs are defined as broadly conserved, functional variability within MAMP domains has been documented. For example, EF-Tu from some bacterial species is not recognized by EFR, due to a single amino acid change in the recognized EF-Tu epitope [34]. The flg22 epitope also exhibits variation between species, or even between strains within a species [30, 40, 43–47]. Relevant to the present study, *Xanthomonas campestris* pv. *campestris* (Xcc) strains such as B305 have a flagellin that is recognized by *Arabidopsis* FLS2 whereas strains such as B186 do not, and the relevant difference is conferred by a single amino acid polymorphism in the flg22 epitope [44]. *Ralstonia solanacearum* strain K60 flagellin contains the amino acid residue necessary for recognition of Xcc strain B305, but differs at other positions of the flg22 region that apparently are responsible for its evasion of recognition [43]. Despite this occasional variability, the recognized MAMP epitopes of proteins do tend to occur in highly conserved regions whose functional constraints and importance to pathogen fitness make them recalcitrant to mutation, e.g., [47, 59, 60, 61]. This general constraint on MAMP domains suggests that slight modifications to MAMP receptors may be sufficient to enable detection of recognition-escaping MAMP variants. There are well-established paradigms for directed in vitro evolution to select for proteins with desired shifts in specificity or other functions [62–66].

Previous study of the β-strand, β-turn face of the FLS2 LRR domain identified the ninth through fifteenth LRR repeats of FLS2 as a likely flg22 binding site, based on the results of *FLS2* mutagenesis and flg22-binding studies [14]. This region of FLS2 was also predicted to be functionally significant by Repeat Conservation Mapping, which uses the primary amino acid sequences of related proteins to find regions of conservation on the modeled surface of folded LRR domains [67]. Recent studies, and especially the co-crystal structure of a FLS2/flg22/BAK1 complex, have provided evidence for several FLS2 extracellular domain sites distributed over many LRR repeats that are required for responsiveness to flg22 [36, 38, 67, 68]. However, the present effort was initiated following the results of [14]. That study utilized thirty-four separate site-directed randomizing mutagenesis libraries, each targeting a single LRR β-strand, β-turn amino acid position in the ninth through fifteenth LRR repeats of FLS2. In most libraries a large majority of the mutated FLS2 receptors still conferred flg22-responsiveness [14]. But in five of the libraries (five amino acid positions), more than 60% of the *FLS2* variants failed to mediate sensitivity to flg22 in transgenic plants even though full-length FLS2 protein was usually detectable [14]. These five amino acid positions are particularly constrained with respect to FLS2 flg22-responsiveness, and inferences that they form a core of the flg22 binding site are supported by the recently published FLS2/flg22/BAK1 crystal structure [36].

In the present study we sought to broaden the flagellin recognition capacity of *Arabidopsis* FLS2. We developed a population of FLS2 receptors mutagenized at locations surrounding the flg22 perception residues predicted by [14]. This FLS2 population was screened for receptors that demonstrated increased sensitivity to the canonical *Pseudomonas aeruginosa* flg22. Positives from this portion of the screen were then tested for enhanced responsiveness to flg22-like peptides designed from plant pathogenic species that wild-type *Arabidopsis* only recognizes weakly or not at all. Although only modest quantitative shifts toward higher peptide sensitivity were obtained, the results indicate that targeted sequence diversification and screening can be a successful approach to the generation of MAMP receptors with potentially desirable changes in ligand specificity.

## Results

### Very low elicitation activity of flg22 peptides from *E. amylovora* and other pathogens

**Fig 1:**
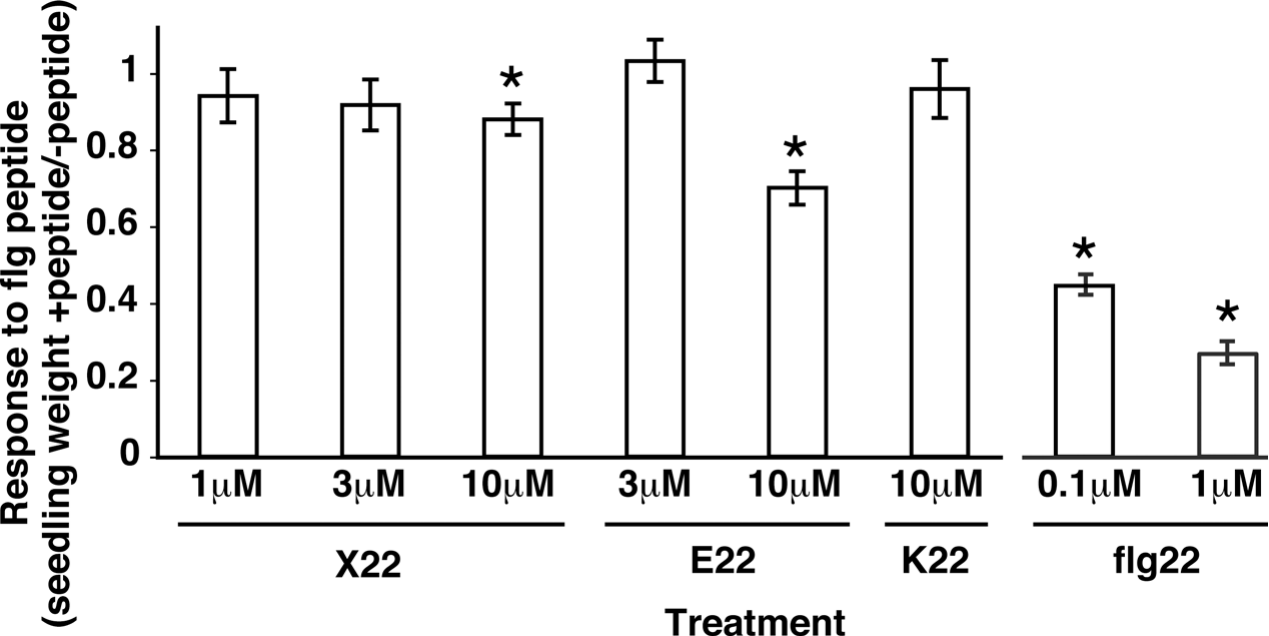
Flg22 regions of three plant pathogens elicit little or no response in *Arabidopsis*. Wild-type *Arabidopsis* (Col-0) plants were tested with the indicated concentrations of peptide in seedling growth inhibition assays. Peptides match the flg22 region of *X*. *campestris* pv. *campestris* strain B186 (X22), *E*. *amylovora* strain CFBP 1430 (E22), *R*. *solanacearum* strain K60 (R22), or canonical flg22 from *P*. *aeruginosa*. Experiment performed three times with n≥8 plants per treatment within each experiment; mean and SE are shown for pooled results; all treatments performed together on same dates except flg22 treatments. Asterisk: significant difference from untreated control plants (ANOVA, p < .05). Note that a hundred-fold lower concentration (0.1 μM) of *P*. *aeruginosa*-based flg22 peptide causes >50% reduction in plant size.

The *Arabidopsis* eliciting activity of flg22 peptides from three plant pathogen isolates, *Ralstonia solanacearum strain* K60, *Xanthomonas campestris* pv. *campestris* strain 186 and *Erwinia amylovora* strain CFBP 1430, was assessed. The peptides flg22_RK60,_ flg22_X186_ and flg22_E1430_ are herein referred to as R22, X22 and E22 respectively; see Table 1. When plant responsiveness to flg peptides was tested using seedling growth inhibition assays, no response of *Arabidopsis* to R22 was detected (Fig 1), as predicted [43]. X22 elicited a slight but statistically significant response only when a very high concentration of peptide (10 μM) was used; no response to X22 could be detected at lower concentrations of peptide (Fig 1). This too was expected; a previous study reported a lack of response of *Arabidopsis* to full-length flagellin extracts from *Xcc* strain 186 [44]. E22 failed to elicit a response except at a concentration of 10 μM (Fig 1). For comparison, the canonical flg22 (based on *P*. *aeruginosa* flagellin) routinely in our laboratory and other laboratories exhibits half-maximal elicitation of *Arabidopsis* responses at concentrations between 10 nM and 100 nM (e.g., Fig 1, subsequent figures, and references [38, 69]).

**Table 1:** Flagellin peptides and sequences

Peptide Name	Species	Sequence
flg22	*Pseudomonas aeruginosa*	QRLSTGSTINSAKDDAAGLQIA
flg22_X186_ (X22)	*Xanthomonas campestris* pv *campestris* strain B186	QQLSSGKRITSASVDAAGLAIS
flg22_E1430_ (E22)	*Erwinia amylovora* strain CFBP 1430	ERLSSGSRITSSKEDAAGQAIS
Flg22_RK60_ (R22)	*Ralstonia solanacearum* strain K60	QRLSTGMRVNSAQDDAAAYASA

### A screen for *FLS2* alleles with elevated sensitivity to flagellin peptides

**Fig 2:**
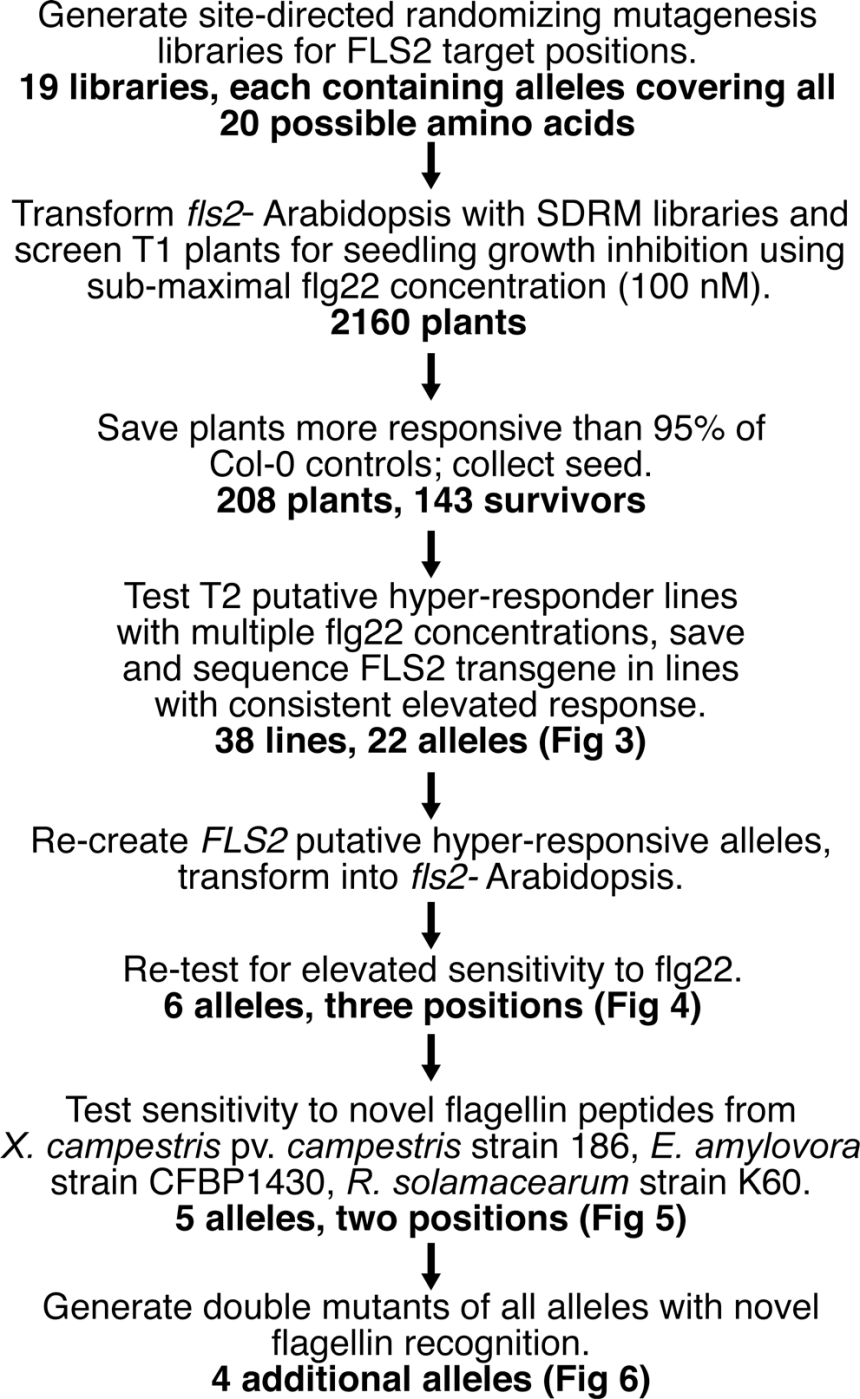
Summary of step-by-step results of screen to identify FLS2 receptors that have increased responsiveness to flg22 peptides.

We attempted to evolve FLS2 towards recognition of previously non-recognized flg22 domains from the above plant pathogenic *Ralstonia*, *Xanthomonas* and *Erwinia* bacteria. This was done by mutagenesis of investigator-chosen amino acid positions in the FLS2 from *Arabidopsis* Col-0, and screening of the resulting alleles in transgenic *Arabidopsis* Col-0 *fls2-101* plants that have a non-functional allele at the native *FLS2* locus. The screen and the results at intermediate steps are summarized in Fig 2. In accord with the findings of other receptor structure-function studies (e.g., [63, 66, 70]), we hypothesized that the five predicted FLS2 solvent-exposed amino acid positions most sensitive to mutation in the study of [14], Arg_294_, Tyr_296_, Gly_318_, Ser_320_, and Asp_414_, are essential for binding/recognition of most or all flagellin peptides, whereas the twenty surface amino acids predicted to be adjacent to these critical amino acids could be positions that contribute to flg22 specificity. We pursued the additional hypothesis that some *FLS2* alleles conferring an elevated sensitivity towards the canonical *P*. *aeruginosa* flg22 would also confer elevated sensitivity to other flg22 peptides. Site-directed randomizing mutagenesis (SDRM) libraries for the twenty residue positions predicted to be spatially adjacent to the above-noted five critical amino acids were therefore tested for increased sensitivity to *P*. *aeruginosa* flg22 (referred to below simply as “flg22”), using a 100 nM concentration of flg22 that elicits a sub-maximal response in wild-type *Arabidopsis* plants in seedling growth inhibition assays. These and all subsequent transgene *FLS2* alleles were constructed to carry the wild-type *FLS2* upstream (promoter) sequence. Increased sensitivity was defined as seedlings that were more responsive than 95% of the approximately 20–120 wild-type controls tested within the same experiment.

**Fig 3:**
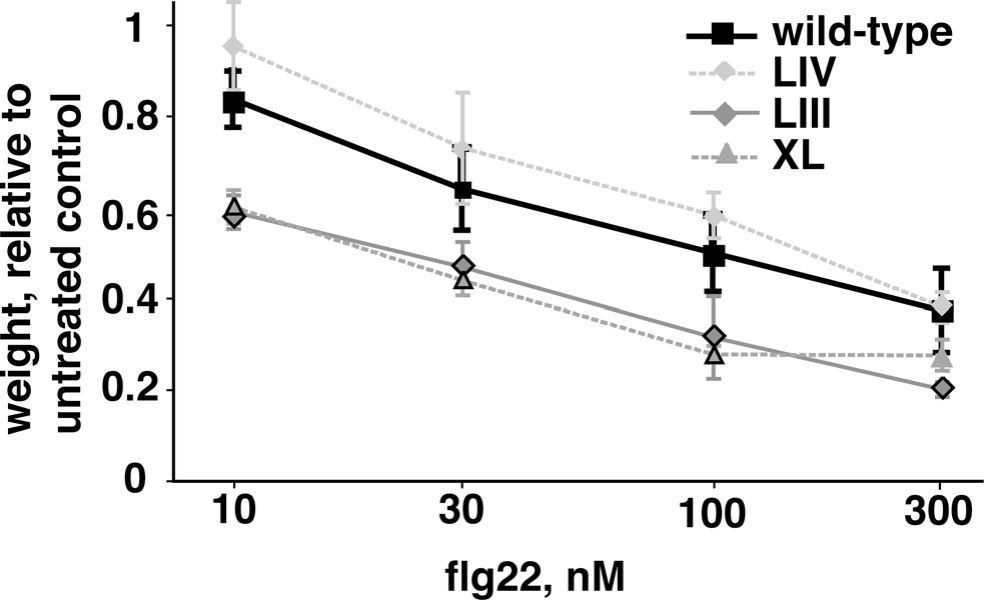
Examples of variant-*FLS2* T_2_ lines that confer elevated sensitivity to flg22. *Arabidopsis* plants (*fls2*^*-*^ mutant background) were transformed with *FLS2* alleles from mutational libraries and T_1_ transgenic plants that were more responsive to canonical flg22 than 95% of wild-type controls were allowed to self-fertilize. T_2_ progeny carrying the vector-associated selectable marker were then tested in a seedling growth inhibition assay of FLS2-mediated response to flg22 peptides. Two example lines that have increased sensitivity to flg22 relative to the wild-type allele (XL and LIII), as well as one example line with no increased sensitivity to any of the flg22 concentrations tested (LIV). Symbols with black outline identify concentrations for a given allele at which plants demonstrated significantly elevated responsiveness to flg22, relative to Col-0 (T-test, P < 0.05). At least eight plants per line per treatment were tested; experiment was repeated twice for each line with similar results.

We screened at least 65 and more typically ~120 clones from each of nineteen SDRM libraries (we were unsuccessful in generating an SDRM library at His_316_, as also reported by [14]). Screening 65 clones from one of these libraries provides an approximately 90% probability of testing all possible amino acid substitutions in that library. Across all nineteen SDRM libraries, 208 candidate plants were identified with possible increased responsiveness to flg22. These plants were saved for seed, and the progeny were re-screened to confirm increased sensitivity to flg22 at a range of concentrations. Of the 143 lines that survived and produced seed, 38 of these progeny lines once again exhibited elevated sensitivity to flg22 for at least one concentration of peptide upon retesting (examples shown in Fig 3). These lines represented 22 novel alleles of *FLS2*, as some of the alleles were recovered two or three times.

Interestingly, 12 of the 38 lines exhibiting elevated flg22 sensitivity had a wild-type *FLS2* sequence. To test the hypothesis that increased expression level of the FLS2 protein was responsible for the increased response to flg22 in these lines, Western blots were performed on whole-cell protein extracts from at least six plants from each line. None of these twelve lines exhibited increased expression of FLS2 protein; in fact, a number of them had lower levels of FLS2 protein than Col-0 controls (examples in S1 Fig). When a subset of these lines carrying an *FLS2* transgene with wild-type sequence was tested for increased response to the novel flagellin peptides R22, X22 and E22, some seemed to exhibit responsiveness (S1 Fig) but upon further testing none reproducibly showed a significantly enhanced response.

**Fig 4:**
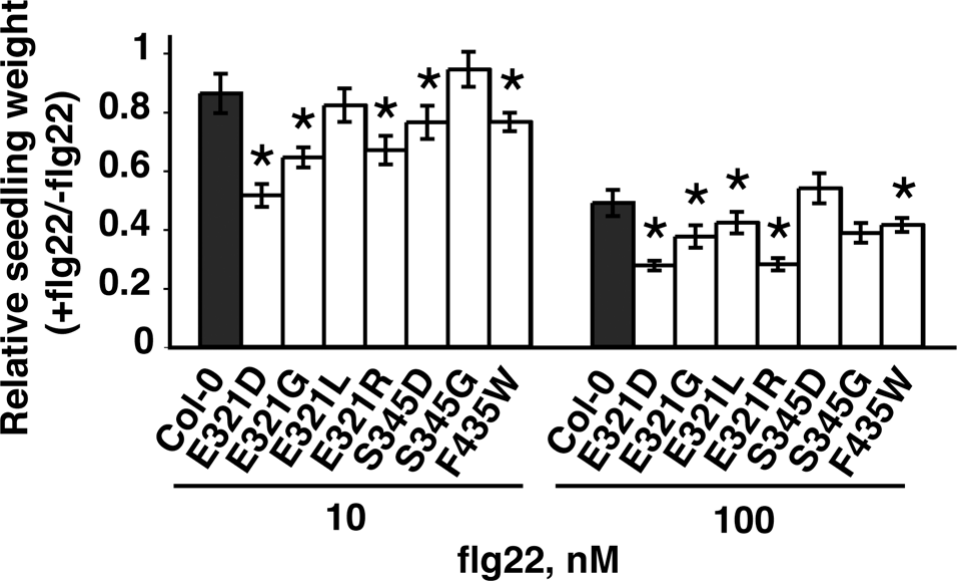
Six alleles at three amino acid positions confer elevated sensitivity to flg22. Re-cloned *FLS2* alleles, derived from all T_2_ lines exhibiting increased flg22 peptide recognition, were re-tested for elevated response to flg22 (n≥8 T_1_ seedlings per genotype per treatment). Results shown for all six alleles that conferred elevated flagellin responsiveness for at least one concentration of flg22, and one allele that did not confer increased recognition of flagellin peptides (S345G). Experiment was performed at least twice per line. Asterisks: significantly different from control plants with a wild-type *FLS2* allele, for that concentration of peptide (ANOVA, p < .05).

Each of the 22 novel alleles of *FLS2* from plant lines in the primary screen that exhibited increased sensitivity towards the canonical flg22 peptide was then independently re-created by site-directed mutagenesis and transformation into *Arabidopsis fls2-101* plants. Multiple independent T_1_ transgenic plants were tested for each construct, to increase the reliability of the test and to expedite progress. When these transgenic plants were screened for elevated sensitivity to *P*. *aeruginosa* flg22, six of these *FLS2* alleles, E321D, E321G, E321L, E321R, S345D, and F435W, conferred elevated responsiveness relative to the wild-type *FLS2* allele, for at least one concentration of flg22, when tested in seedling growth inhibition assays (Fig 4; for dose response curves across several flg22 concentrations see S2 Fig).

### A subset of *FLS2* alleles with increased recognition of flg22 also confer increased recognition of a flagellin peptide from *E*. *amylovora*

**Fig 5:**
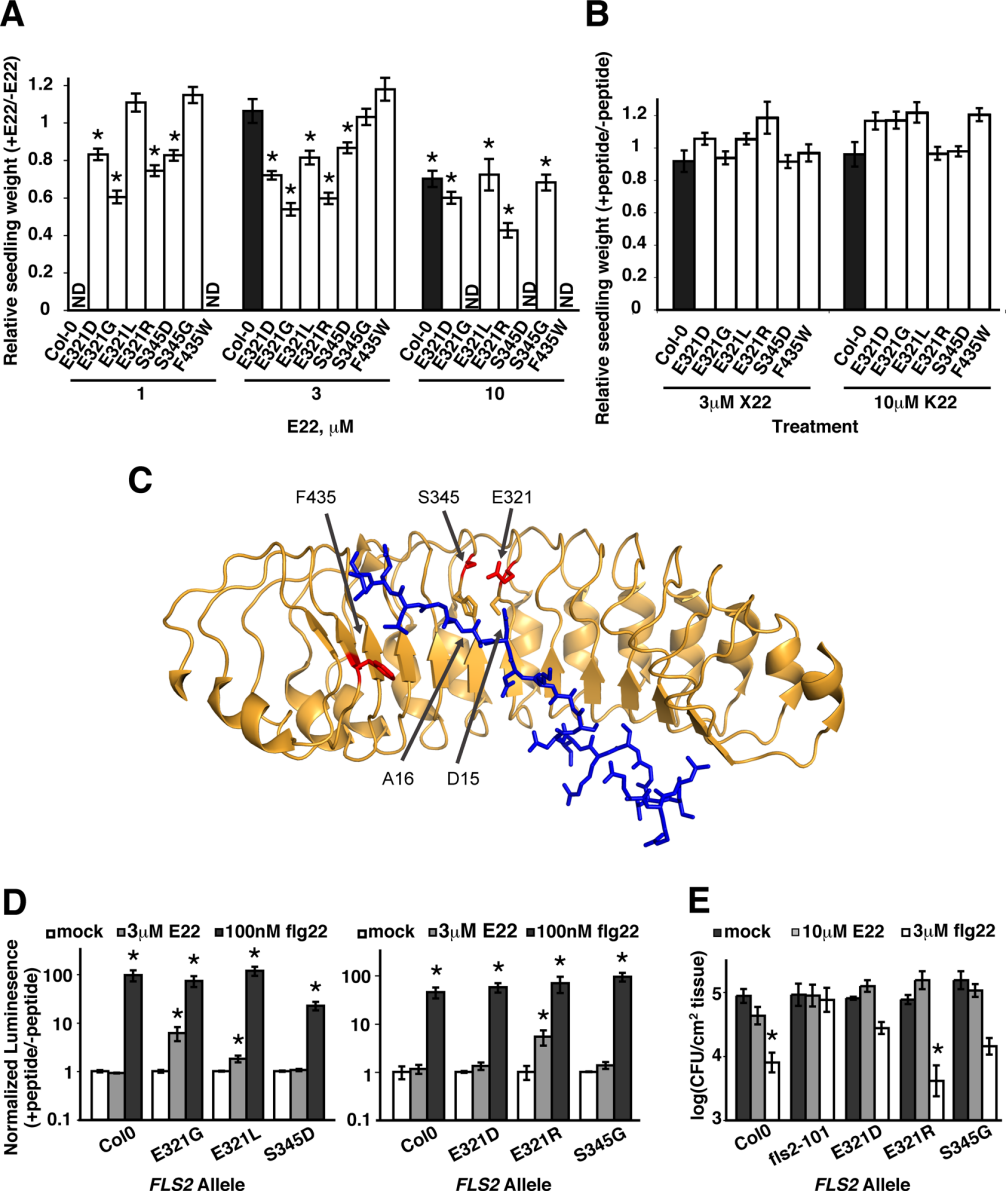
Five elevated-flg22-response alleles of *FLS2* identify two amino acid positions that confer increased recognition of *Erwinia amylovora* flg22 (E22). All bars present pooled data from two to three independent experiments. (**A**) Seedling growth inhibition assay of FLS2-mediated response to flg peptides. At least eight T_1_ plants per treatment per genotype were tested. Asterisks: significant differences of E22-treated samples from mock-treated samples within a given genotype (ANOVA, p < .05); ND: not determined. (**B**) Response to X22 and E22 peptides in a seedling growth inhibition assay. At least eight T_1_ plants per treatment per genotype were tested. None of the observed responses were significantly different from mock-treated samples of the same genotype (ANOVA, all p>.05). (**C**) Location of FLS2 residues relative to flg22 residues in FLS2/flg22/BAK1 co-crystal structure of [36]. Tan: FLS2 LRR main chain (cartoon representation; arrows = beta-strand regions; twelve repeats of the LRR are shown; amino-terminus to right and juxtamembrane region to left in picture); red: wild-type E321, S345 and F435 residues that were mutated in elevated-response FLS2 proteins; tan side-chains: adjacent S320 and H344 residues that bind flg22; blue: flg22 main chain and side chains in stick representation (flg22 carboxy-terminus is in upper left). Portions of BAK1, FLS2 and an SO_4_ from co-crystal structure would occupy foreground in upper left and are removed from view. (**D**) ROS burst in response to peptides. Area under curve for 30 min. ROS burst for the designated allele and treatment divided by mean area for mock-treated leaf discs of same genotype in same experiment. Assays performed on different days are shown in separate graphs. Asterisks: significantly more total ROS production relative to mock-treated controls within a given genotype (ANOVA, p < .05). (**E**) Bacterial growth assays using in six-week old T_2_ plants containing receptors of interest (as well as Col-0 and *fls2*^*-*^ controls). Rosette leaves were infiltrated with the indicated peptides 24 hours before infiltration with 10^5^ cfu/mL *P*. *syringae* pv. *tomato* DC3000. Two days later, leaf samples were removed and bacterial titers were determined.

When tested for their response to R22, X22, and E22, five of the above six *FLS2* alleles (all but F435W) also conferred recognition of 3 μM E22 in a seedling growth inhibition assay, whereas a wild-type *FLS2* allele does not confer response to this concentration of E22 (Fig 4A). Four of these new alleles were also responsive to 1 μM E22 (Fig 5A). The sixteen re-cloned *FLS2* alleles that did not have elevated sensitivity to flg22 also did not respond to any of the tested flagellin peptides (Fig 5A and data not shown). For alleles that did confer a response to E22, the magnitude was markedly less than the response to flg22 (wild-type seedlings grown in the presence of 1 μM flg22 are approximately 25% the fresh weight of their non-treated counterparts (e.g., Figs 1, 3 and 4B) whereas plants responding to 3 μM E22 were, at best, approximately 50% the fresh weight of the untreated control). None of the 22 re-cloned *FLS2* alleles, including the six elevated-response alleles, conferred recognition of 10 μM R22 or 3 μM X22 in a seedling growth inhibition assay (Fig 5B and data not shown).

Fig 5C shows the location of the E321 and S345 residues of FLS2, relative to the site at which flg22 bound to FLS2 in the FLS2/flg22/BAK1 co-crystal structure of [36]. E321 and S345 are adjacent to the S320 and H344 residues that form part of the flg22 binding site, and the proximity of acidic E321 to flg22 acidic residue D15 suggests likely impacts on flg22-FLS2 interaction (see Discussion). The F435W mutation (that caused elevated response to flg22 but not to E22, R22 or X22) is located, together with FLS2 residue L412, at the interaction site with the side chain of flg22 residue L19 in the FLS2/flg22/BAK1 co-crystal structure (not shown). FLS2 residues E321, S345 and F435 do not contact and are not adjacent to FLS2 residues that contact BAK1 in the FLS2/flg22/BAK1 co-crystal structure [36].

We then conducted reactive oxygen species (ROS) burst and *P*. *syringae* growth assays to further characterize the immune response mediated by the novel *FLS2* alleles. Plants (*fls2* mutant background) expressing any of the three alleles identified as most E22-responsive in a seedling growth inhibition assay (E321G, E321L, and E321R) generated a ROS burst in response to 3μM E22, but Col-0 control plants did not (Fig 5D; ROS time course traces shown in S3 Fig). No ROS response to E22 was observed in plants carrying the E321D, S345D or S345G alleles, possibly due to the lower sensitivity of the ROS assay relative to the seedling growth inhibition assay. All alleles tested could confer an ROS response to the canonical flg22 peptide (Fig 5D).

Having confirmed responsiveness to E22, we then tested selected *FLS2* alleles to see if the moderate, quantitatively partial response they conferred was sufficient to constrain the growth of virulent *P*. *syringae* pv. *tomato* strain DC3000. Plants were pre-treated with E22 or the canonical flg22 peptide and then, one day later, challenged by infiltration of DC3000 directly into leaves. As previously reported (e.g., [12, 44]), wild-type Col-0 plants pre-treated with flg22 exhibited approximately a 10-fold reduction in the growth of DC3000 in leaves, and as expected, no reduction in DC3000 growth was observed when wild-type plants were treated with 10μM E22 (Fig 5E). The two elevated-response *FLS2* alleles tested in this assay included E321R, the most robust responder in ROS and seedling growth inhibition assays. *Arabidopsis fls2-101* plants transgenic for the new *FLS2* alleles did not display increased resistance to DC3000 following pre-treatment with E22, although pre-treatment of the same genotypes with flg22 was protective (Fig 5E).

**Fig 6:**
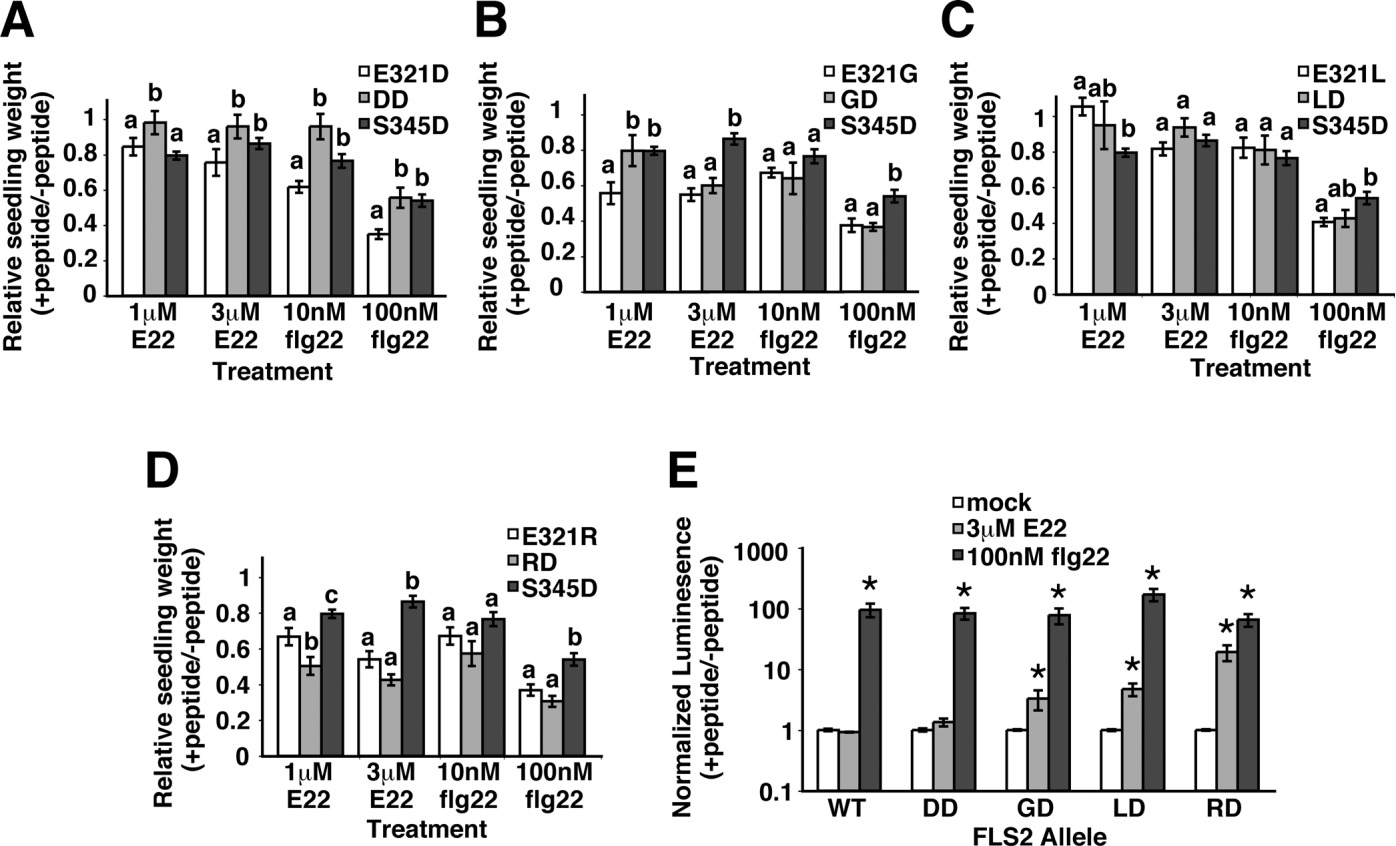
Sensitivity of *FLS2* alleles with two amino acid mutations that confer recognition of *E*. *amylovora* flagellin peptide. Seedling growth inhibition assays of FLS2-mediated response to flg peptides, performed with the indicated peptides using T_1_ plants (*fls2*^*-*^ background) transformed with the *FLS2* constructs (**A**) E321D/S345D, (**B)** E321G/S345D, (**C**) E321L/S345D, and (**D**) E321R/S345D, or with the corresponding single-mutation *FLS2* alleles. Within each set of three bars, *FLS2* alleles giving a significantly different response to the indicated peptide have different letters (ANOVA, p < .05). Experiments repeated at least two times; n≥7 (typically n = 10 or 14) T_1_ seedlings per genotype per treatment in each experiment. (**E**) ROS burst response mediated by the four possible double-mutant *FLS2* alleles, normalized to average of untreated controls from the same day. Experiments repeated at least two times; pooled data are presented as mean and SE for area under luminescence curve for 30 min. ROS burst. Asterisks: significantly more ROS than mock-treated samples (ANOVA, p < .05).

In subsequent work, experiments were performed to determine if the effects of the selected mutations could be additive. All four possible double mutation *FLS2* alleles were made based on the single-mutation *FLS2* alleles that conferred recognition of E22. Multiple independent transformants were then tested for each construct (Fig 6). Three allele combinations (E321D/S345D, E321G/S345D and E321L/S345D) did not increase sensitivity to E22 or flg22 in seedling growth inhibition assays; these double alleles conferred sensitivity to E22 and flg22 that was similar to or weaker than their respective single alleles (Fig 6A–6C and 6E). However, the double mutant E321R/S345D did provide significantly increased recognition of 1 μM E22 peptide (Fig 6D). The double mutant *FLS2* alleles were further tested using ROS assays. Once again, the E321R/S345D allele conferred the strongest observed response to E22 peptide (Fig 6E; y-axis is logarithmic scale). The results suggest the feasibility of gaining enhanced responsiveness to poorly recognized flagellin variants by identifying and then combining FLS2 mutations that individually confer only modest increases in flagellin sensitivity.

## Discussion

MAMP perception is an important component of plant immunity. While plants are able to recognize a diverse repertoire of molecules from microbes, several plant pathogenic species have evolved such that some of their MAMPs are no longer detectable by the immune systems of some plants. We set out to evolve the FLS2 flagellin receptor of *Arabidopsis* towards recognition of flagellin peptides from three plant pathogen isolates that *Arabidopsis* FLS2 currently cannot recognize: *X*. *campestris* pv. *campestris* strain B186, *E*. *amylovora* strain CFBP 1430, and *R*. *solanacearum* strain K60. An *FLS2* allele library was generated that carries mutations in residues directly adjacent to residues previously implicated in flagellin recognition. Our approach explored the hypothesis that screening of *FLS2* alleles for elevated response to a known high-affinity ligand (canonical *P*. *aeruginosa*-based flg22 peptide) could be used to form a foundation for generation of receptor variants with elevated responsiveness to less sensitively detected ligands (such as the E22 *E*. *amylovora*-based flg22 peptide). Obvious approaches for future screens could include conducting the initial screen directly with the poorly recognized target peptide, and/or mutagenizing the receptor residues that directly interact with the ligand. However, the peptides X22, R22 and E22 (Table 1) are significantly different from flg22 and a more efficient interaction with these peptides might cause decreased rather than increased responsiveness to commonly present flg22 regions of other bacterial pathogens. The flagellin flg22 regions that are efficiently recognized by current wild-type FLS2 receptors presumably represent the prevailing evolutionary selection that has shaped FLS2 specificity—a recognition capacity that it is important to retain. Nevertheless, ectopic expression of revised-affinity FLS2 receptors in plants that also retain endogenous wild-type FLS2 receptors might be a successful approach to expand the flagellin detection capacity of plant immune systems without sacrificing existing flagellin detection capabilities.

In the primary screen of the present study, it was surprising that twelve of the thirty-eight lines of plants with increased responsiveness to flg22 had a wild-type FLS2 sequence. We initially hypothesized that this elevated sensitivity was due to increased expression of the FLS2 protein–a hypothesis supported by other recent work [71]. However, FLS2 protein levels were similar to or lower than wild-type in the lines with increased responsiveness to flg22 that carried a wild-type *FLS2* transgene sequence. Additionally, sixteen novel alleles from lines that had elevated sensitivity to flg22 did not confer this increased responsiveness to flg22 when re-transformed. Because we defined ‘elevated-responsiveness’ candidates to be those that fell outside the range of 95% of all wild-type seedlings tested on a particular day, it was expected that a substantial number of the plant lines recovered would be false positives rather than true elevated responders. However, for some of the alleles that did not reproducibly elevate response to flg22 in subsequently generated allele re-test transformants, results with progeny of the original ‘elevated-responsiveness’ transformants suggested that a true elevation of responsiveness to flg22 had occurred in the original transformed lines independent of the encoded FLS2 sequence. It is possible that the insertion sites of the transgene constructs were the cause of this phenotype, or alteration of epigenetic marks. The work of [71] explicitly suggests that in addition to variation in FLS2 expression levels, variation in the signaling pathways downstream of MAMP receptors is likely to contribute to observed variations in flagellin perception. The transgenic plant lines we isolated that exhibited elevated responsiveness yet contained uninteresting *FLS2* alleles may have arisen due to altered epigenetic regulation of other regulators of FLS2 signaling; plant lines with these characteristics may be of interest for future study.

Four of the six single amino acid change *FLS2* alleles identified here that confer increased responsiveness to flagellin peptides contained mutations at the same amino acid residue, E321. Furthermore, the properties of the four amino acids in these alleles (glycine, aspartic acid, leucine, and arginine) are markedly different from one another in terms of physical and chemical properties. One possible explanation for this observation is that the wild-type glutamate residue at this position serves as a constraining factor in flg22 recognition, and removal of this amino acid allowed for more promiscuous binding by the receptor. This could be caused by removal of the relatively large, negative amino acid and replacement either by a smaller amino acid (in the case of glycine, aspartic acid, or leucine) or a positively charged amino acid (in the case of arginine). In the FLS2/flg22/BAK1 co-crystal structure [36], and as shown in Fig 5C, FLS2 residues E321 and especially S320 are adjacent to D15 of the flg22 peptide (D15 of flg22 = D44 of commonly referenced *Salmonella enterica* flagellin crystal structure 1UCU; [72]). Hence the FLS2/flg22/BAK1 co-crystal structure supports this hypothesized repulsive effect between wild-type FLS2 glutamic acid residue 321 and the flg22 aspartic acid residue D15, which is conserved across all four of the flagellins used in the present study (Table 1). The enhanced recognition of the *Erwinia amylovora*-based E22 peptide by the FLS2 E321 mutants, without detectably elevated recognition of the R22 or X22 peptides, may be due in part to the larger size of the E14 acidic side chain in the E22 peptide relative to the D14 residue in flg22 and R22 and the V14 residue in X22. The E14 residue in E22 may force D15 of the E22 peptide into closer proximity with FLS2 residue E321.

We did not detect a response to the E22 peptide in all types of assays performed. The ROS assay only revealed elevated responses to E22 peptide for three of the six alleles that reproducibly conferred a response to E22 in seedling growth inhibition assays, and none of the responses to E22 was strong enough to activate detectable restriction of *P*. *syringae* pv. *tomato* strain DC3000 bacterial growth. Recent studies have indicated that some MAMP response assays give conflicting results depending on lab-to-lab variation in specific plant culture environment variables [73]. Other work has shown that MAMP receptors do not uniformly or universally activate all of the characteristic downstream responses (e.g., [10, 74, 75, 76]). Our ROS and pathogen restriction assays may not have been performed in conditions that optimize those responses, or, the modest response to E22 conferred by the new *FLS2* alleles may be sufficient to surpass a threshold for triggering only some and not all FLS2-mediated plant responses. It was encouraging, however, that testing of only four second-round *FLS2* alleles (all pairwise combinations of the E321 and S345 mutations) yielded an allele that mediated even stronger seedling growth inhibition and ROS burst responses to the E22 *Erwinia amylovora* flagellin segment.

In the future, it may be possible to enhance the function of insufficiently active MAMP receptors by a number of approaches. Random in vitro mutagenesis of NLR protein LRR domains (and other domains) has, in a few cases, been used to extend the pathogen recognition specificity of those proteins [77–82]. Iterative rounds of further modification and screening are the standard approach to protein improvement by in vitro evolution [62, 64, 66, 70, 78]. For PRRs, creation of chimeric receptors with a recognition domain fused to a highly active catalytic domain might increase the response to weakly recognized peptides. Functional chimeric MAMP receptors have been demonstrated [83–85]. Elevated efficacy might also be engineered by improved expression of receptors through increased transcription, more efficient protein processing in the ER, decreased protein turnover, elevated affinity for co-receptor proteins, or other mechanisms [71, 86]. ER quality control of MAMP receptors is known to be an important means of regulating expression of these receptors and therefore response to MAMPs [87–89]. Protein modifications such as glycosylation also have been shown to be essential for some MAMP receptors including EFR and should be considered when optimizing MAMP receptor performance, although they apparently make a less substantial contribution to FLS2 [90, 91]. A duplication and divergence strategy could also broaden the spectrum of resistance, pyramiding expression of multiple naturally occurring or engineered MAMP receptors that are substantially similar but which recognize a more diverse range of ligands.

Our screen succeeded in identifying mutations that increase sensitivity to a previously unrecognized flagellin peptide, but increasing the throughput of receptor screening would clearly allow searches of wider pools of receptors and more rapid identification of such mutants. For example, although our efforts with yeast to date have not yielded display of FLS2 or EFR extracellular domains that exhibit expected ligand specificity, yeast cell surface display [92] of modified FLS2 or EFR domains could allow functional screening of millions of different alleles. However, yeast display and other methods may be difficult to optimize if for example appropriate MAMP docking relies on other plant cofactors or co-receptors (such as BAK1; [36]), or plant-specific receptor glycosylation. Iterative rounds of mutagenesis and selection, that perform as desired and successfully produce higher-affinity and/or altered-specificity MAMP receptors in yeast, also may or may not lead to receptors that improve disease resistance or overall plant performance when moved back into plants. The *in planta* experiments presented here, although low-throughput, are achievable with present technology and are more likely to provide biologically relevant data. Higher-throughput *in planta* selection systems may represent the optimum system for future work.

We chose flagellin sequences from *X*. *campestris* pv. *campestris*, *E*. *amylovora*, and *R*. *solanacearum* as the targets for our studies, but it should be possible to modify the methods utilized here to target other pathogens or MAMPs. For example, there are elf18 peptides from certain plant pathogenic species that cannot be recognized by EFR [34] and an analogous approach to the one taken here might expand EFR recognition toward such ligands. With ongoing refinement, these approaches may in the future allow guided increases in the plant immune receptor arsenal.

## Materials and Methods

### Plants and bacterial growth conditions

*Arabidopsis thaliana* Col-0 and Col-0 *fls2-101* plant lines have been previously described [43]. Plants were grown in soil-less potting mix (Sunshine Mix 1, SunGro Horticulture, Agawam MA USA) in nine-hour days, or in fifteen-hour days for plants grown on agar or in liquid half-strength MS plus 1% sucrose. *P*. *syringae* pv. *tomato* strain DC3000 carrying the empty cloning vector pVSP61 [93] was grown at 28°C on NYGA containing 25 μg/mL kanamycin and 50 μg/mL rifampicin.

### Cloning and plant transformation

Site-directed randomizing mutagenesis (SDRM) libraries were previously reported [14, 67]. Briefly, the LRR from *Arabidopsis thaliana FLS2* from accession Col-0, with added AscI and PacI restriction sites, was cloned into pENTR/D-TOPO (Invitrogen) and used as the template for SDRM. Mutagenic primers containing a randomizing codon at the site of interest (NNB, where N is any nucleotide and B is any nucleotide but A) were used for PCR with a high-fidelity Taq polymerase (Pfu Turbo, Stratagene, La Jolla, CA, USA). Template was digested with the methylation-sensitive restriction endonuclease DpnI, and products were transformed into *E*. *coli*. Libraries were verified by sequencing of multiple individual clones and subsequently cut and pasted into the binary vector pHD3300 and pHD3300HA, which contain 1kb of native *FLS2* promoter, and the rest of the FLS2 ORF containing the engineered AscI and PacI sites without or with an HA tag, respectively [14]. Mutated FLS2 LRR regions from pENTR/D-TOPO were moved into these vectors by digestion of both vectors with AscI and PacI and subsequent ligation. Stable transformants of *Arabidopsis* Col-0 *fls2-101* plants (containing a premature stop codon in *FLS2* open reading frame [43]) were generated via dipping with *Agrobacterium tumefaciens* GV3101 (pMP90) carrying the construct of interest. *FLS2* alleles with multiple amino acids more than a few residues apart were generated sequentially using this strategy, using template containing some of the desired mutations and primers that would generate the additional mutations.

To determine the *FLS2* transgene allele in lines with elevated flg22 sensitivity, DNA was extracted and used as a template to amplify the *FLS2* LRR-encoding domain (using primers CAGATTGGACCATGATCGGTTCGGCGCGCC and CCCATTAGATCAGAGGCGTTAATTAA, which are specific for the AscI and PacI sites in the transgene and not in the endogenous, mutationally inactivated *fls2-101* allele of the host plant), and the DNA sequence of the PCR product was then determined. In sequences from plants with elevated responsiveness to flg22 that had a wild-type sequence, the entire ORF of *FLS2* was sequenced to check for the presence of mutations outside of the LRR domain.

### Protein detection

Six to eight three-week-old seedlings were ground at room temperature in 2x SDS buffer (2mL buffer per g tissue), boiled, and centrifuged to remove particulates. 50 μl per sample was separated by SDS-PAGE, blotted onto a PVDF membrane, and detected using a rabbit anti-FLS2 antibody (Genscript, Piscataway NJ USA; prepared as in [94]) that was subsequently detected by a goat IgG anti-rabbit antibody conjugated to horseradish peroxidase (Roche, Indianapolis IN USA), made visible using the ECL Plus Kit (Amersham/GE Healthcare, Pittsburgh PA USA).

### Peptide response assays

Flagellin peptides used are listed in Table 1 and were obtained from Genscript.

Seedling growth inhibition assays were performed as in [14, 16, 67]. Briefly, seedlings were grown on half-strength MS with relevant selection for five days. Transgenic seedlings were then moved to liquid half-strength MS plus 1% sucrose with indicated concentration of flg22 peptide and grown for 10–14 days before being weighed. For any individual experiment, all seedlings were weighed on the same day.

Reactive oxygen species (ROS) were detected as described previously [15, 67]. Briefly, leaf discs were taken from leaves of four- to eight-week old stable transgenic *Arabidopsis* plants containing our construct of interest. Leaf discs were floated on 1% DMSO overnight before being treated with indicated peptides in the presence of 1 μg/mL luminol and 1 μg/mL horseradish peroxidase. Luminescence was measured on a Synergy HT or a Berthold Centro microplate reader for 30 minutes immediately following addition of reagents. At least two T_2_ plants per construct and four leaf discs per treatment per plant were used in each replicate.

For bacterial growth assays, leaves of five- to six-week old plants were infiltrated via syringe with 10mM MgCl_2_ with the indicated concentration of flagellin peptide. 24 hours later, the same leaves were infiltrated with *P*. *syringae* pv. *tomato* strain DC3000(pVSP61) at a concentration of 10^5^ CFU/mL. Two days later, four leaf discs from two plants (two leaves per plant) were ground in 10mM MgCl_2_ and dilution-plated on NYGA containing cycloheximide (antifungal) and rifampicin to determine bacteria levels for a single replicate. Each genotype/treatment combination was tested in triplicate. Colonies were counted two days later for enumeration of leaf bacteria.

Statistical tests (ANOVA with Tukey’s multiple comparison test) were performed using Minitab.

## Supporting Information

S1 Fig Characterization of *fls2*^*-*^ Arabidopsis lines, carrying an *FLS2* transgene with wild-type sequence, that exhibited increased sensitivity to flg22. (PDF)

S2 Fig Six alleles at three amino acid positions confer elevated sensitivity to flg22. (PDF)

S3 Fig ROS burst in response to *Erwinia amylovora* flg22 (E22) peptide conferred by some elevated-flg22-response alleles of *FLS2*. (PDF)
