# Supplementary material for: Directed Evolution of FLS2 towards Novel Flagellin Peptide Recognition
Source: PLoS One. 2016 Jun 6;11(6):e0157155. doi: 10.1371/journal.pone.0157155 (PMC4894583; doi:10.1371/journal.pone.0157155)
Supplement: S1 Fig — (PDF) [file pone.0157155.s001.pdf]

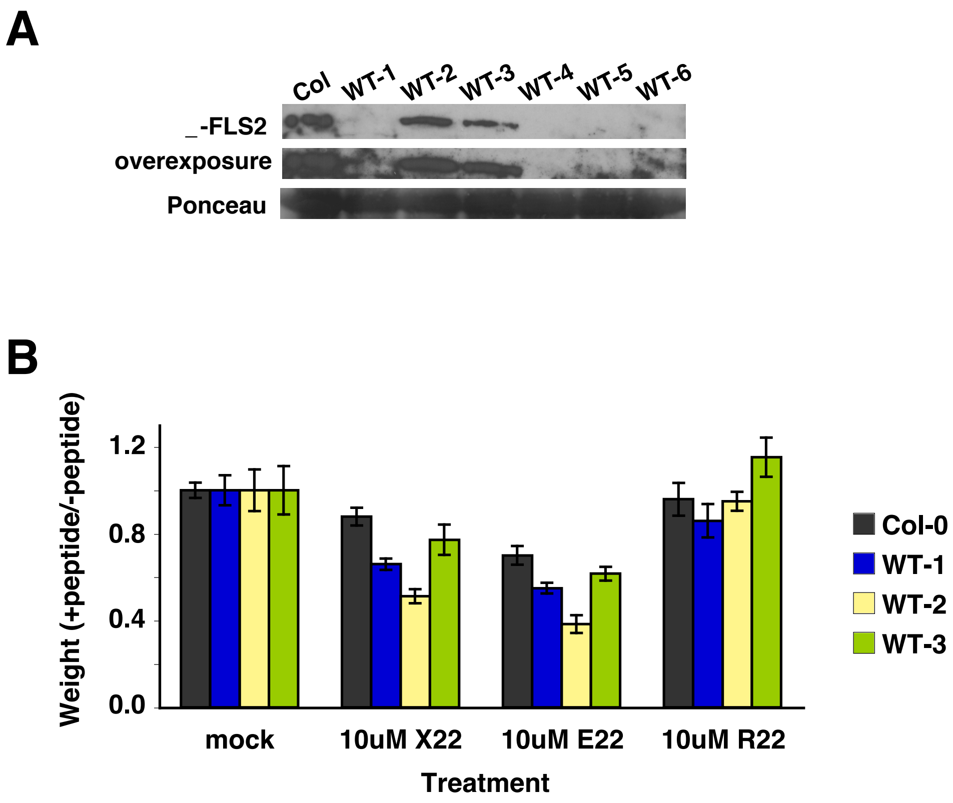

S1 Figure: Characterization of *fls2*$^{-}$ Arabidopsis lines, carrying an *FLS2* transgene with wild-type sequence, that exhibited increased sensitivity to flg22. (A) Expression of FLS2 protein in these lines. **(B)** Response to novel peptides in a seeding growth inhibition assay (in subsequent testing this response was not reproducibly detected).
