# Supplementary material for: Directed Evolution of FLS2 towards Novel Flagellin Peptide Recognition
Source: PLoS One. 2016 Jun 6;11(6):e0157155. doi: 10.1371/journal.pone.0157155 (PMC4894583; doi:10.1371/journal.pone.0157155)
Supplement: S2 Fig — (PDF) [file pone.0157155.s002.pdf]

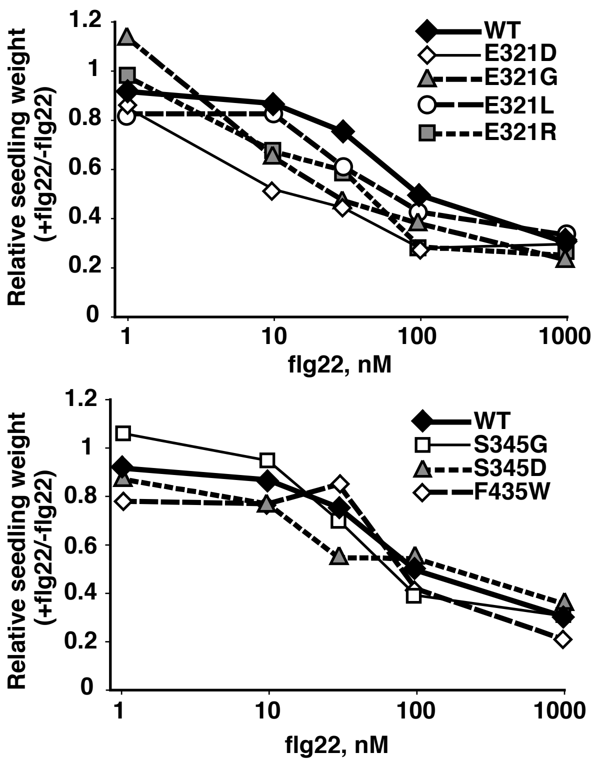

S2 Figure: Six alleles at three amino acid positions confer elevated sensitivity to flg22. See also Fig 4, which presents same data for 10 nM and 100nM treatments, in a different format. Re-cloned *FLS2* alleles, derived from all T2 lines exhibiting increased flg22 peptide recognition, were re-tested for elevated response to flg22 (n>7 T1 seedlings per genotype per treatment). Results shown for all six alleles that conferred elevated flagellin responsiveness at at least one concentration of flg22 and one allele that did not confer increased recognition of flagellin peptides (S345G). Experiment was performed at least twice per line.
