# Supplementary material for: Directed Evolution of FLS2 towards Novel Flagellin Peptide Recognition
Source: PLoS One. 2016 Jun 6;11(6):e0157155. doi: 10.1371/journal.pone.0157155 (PMC4894583; doi:10.1371/journal.pone.0157155)
Supplement: S3 Fig — (PDF) [file pone.0157155.s003.pdf]

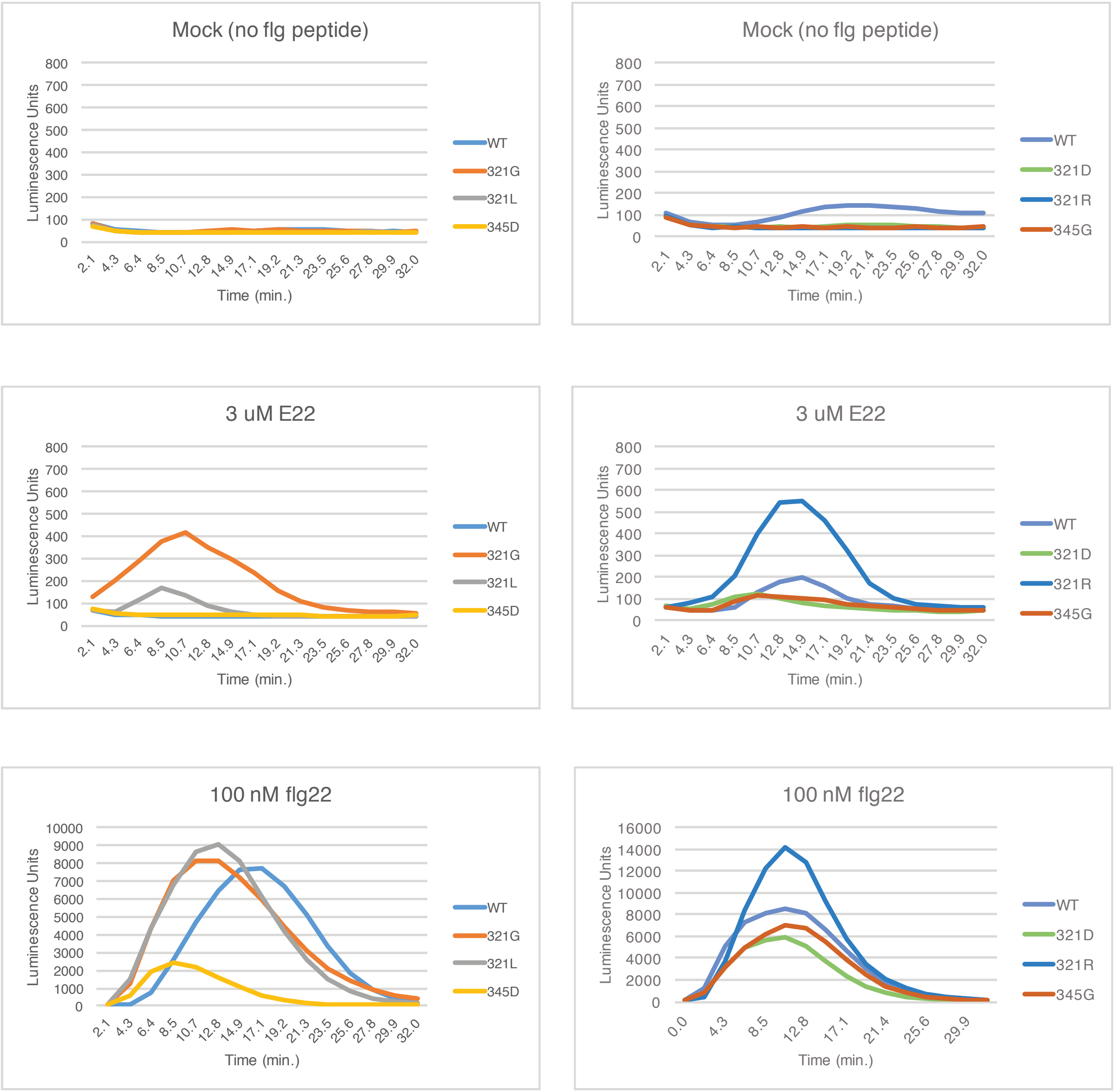


**S3 Figure: ROS burst in response to *Erwinia amylovora* flg22 (E22) peptide conferred by some elevated-flg22-response alleles of *FLS2*.** Same dataset as Fig 5D, but showing ROS burst traces over time. Peptide treatment is given at top of graph, *FLS2* allele noted on right side. Each time point on each trace is the average of eight separate leaf discs that received same treatment, pooled from two separate experiments. Experiments in left column of graphs performed together on same dates. Experiments in right column of graphs performed together on same dates, but on separate dates from left column of graphs.
